# Recurring outbreaks by the same *Escherichia coli* ST10 clone in a broiler unit during 18 months

**DOI:** 10.1186/s13567-021-01017-6

**Published:** 2022-01-09

**Authors:** Anders Miki Bojesen, Umran Ahmed, Hanne Skaarup, Carmen Espinosa-Gongora

**Affiliations:** 1 Department of Veterinary and Animal Sciences, University of Copenhagen, Frederiksberg C, Denmark; 2 Danhatch Denmark, Vrå, Denmark

**Keywords:** multiple outbreaks, broiler, *E. coli* ST10, clonality

## Abstract

The current investigation aimed at characterizing the cause of multiple disease outbreaks in the same broiler production unit during a course of 18 months. The outbreaks had mortality rates of up to 22%. *Escherichia coli* was diagnosed as the responsible agent. Multiple-locus variable-number tandem-repeat analysis showed that all chicken isolates had identical band patterns. Core genome comparisons demonstrated that the 36 chicken isolates differed with maximum of nine nucleotides indicating that the same *E. coli* clone was responsible for all seven disease outbreaks despite adherence to the all-in-all production principle and rigorous cleaning and disinfection procedures.

**Supplementary Information:**

The online version contains supplementary material available at 10.1186/s13567-021-01017-6.

## Introduction, methods and results

Modern broiler production relies on complete separation of chickens of different generations and ages, so as to prevent transmission of pathogens between groups of individuals with differences in immunity towards common infectious agents. Much emphasis has been placed on keeping high biosecurity measures to protect vulnerable chickens against microorganisms in the chicken feed, water and air intakes of the production units. While horizontal spread of pathogens from the surrounding environment or littermates is a well-described route of transmission, vertical transfer from the parents to the offspring through the hatching egg receives increasing attention [1]. *Escherichia coli*, which by far, is the most frequent bacterial cause of infectious disease in broilers is able of both vertical and horizontal transfer [2]. In the event of a disease outbreak, tracking of the source begins immediately, particularly if the problem seems to reoccur.

Recent advances in genotypic characterization methods have allowed the use of increasingly refined techniques to establish epidemiological connections and probabilities. Rapid and accurate PCR-based methods and particularly analyses based on whole genome sequences (WGS) have become comparably competitive in price. WGS not only allows in-depth analysis of genetic relatedness but also enables insight into presence of antimicrobial resistance genes, genes encoding virulence factors and other determinants of interest. Freely available online tools like Enterobase [3] and CSI Phylogeny [4] allows user-friendly access to databases and sequence data analysis resources with high practical value.

The current report originates from a broiler farm unit, built in 1992 and holding up to 40 000 chickens at one time. The production followed the “all in–all out” principles. After each production cycle, or rotation, a cleaning and disinfection procedure including complete removal of all litter prior to cleaning with water was performed. The disinfection procedure included sweeping of all surfaces inside the unit with Virucid™ (mix of quaternary ammonium compounds, Didecyldimethylammonium chloride, Glutaraldehyde and Propan-2-ol) and by fumigation according to the manufactures instructions (CID Lines NV, Leper, Belgium). After every second rotation the floor was treated with a caustic soda solution (Brenntag Nordic, Ballerup, Denmark). The drinking water lines were cleaned and disinfected with CID 2000 according to the manufactures instructions (CID Lines NV, Leper, Belgium). Following a 7–10-day empty period, new bedding material was added, and a new batch of day-old chickens was transferred from the hatchery. The unit thus passed approx. 7–8 rotations of broilers per year. The broilers were Ross308 sourced from parent’s stock farms delivering hatching eggs to Danhatch Denmark’s hatcheries in Vrå (Northern Jutland, Denmark) and Ragebøl (Southern Jutland, Denmark), respectively. During an 18-month period, starting June 2017 and ending November 2018, eight out of 11 rotations experienced a significantly increased mortality rate (*p*  < 0.00009) during the approx. 35-day production period, when compared to the 11 subsequent non-affected cycles (Table 1). Mortality reached as high as 1% per day, and more than 20% accumulated mortality was recorded in some rotations. The first-week mortality did not differ statistically between the groups compared (*p*  = 0.16), nor did any of the other characteristics investigated.

**Table 1:** **Production data for 11 rotations affected by high mortality and 11 subsequent rotations not affected by high mortality.**

	First week mortality (%)	Total mortality (%)	Weight first-week (g)	Weight at slaughter (g)	Age at slaughter (d)	Condemnation at slaughter (%)		No. of chicks per rotation
Affected rotations								
Mean	1.6	13.1^a^	173.5	2247	34.4	1.4	34 150	
Stdv	2.2	5.3	15.8	221	1.7	0.4	1870	
Range	0.6–7.8	6.1–21.7	150–210	1693–2523	31.1–37.6	0.7–1.8	32 500–39 000	
Non-affected rotations								
Mean	0.6	2.7	170.8	2128	34.9	1.9	33 680	
Stdv	0.3	0.9	7.1	232	1.8	2.7	2448	
Range	0.4–1.2	1.5–4.5	160–180	1735–2474	32.2–37.3	0.4–9.4	29 000–37 000	

^a^The total mortality rate was significantly higher (T test) in the *E. coli* outbreak-affected rotations (*p*  < 0.00009).

Common to all outbreaks was a sudden onset of clinical signs including depression, inappetence, reluctance to move and a steep rise in mortality. The affected chickens had generally passed the first week of life, which is a typical period for *E. coli* infections following vertical transmission. Dead chickens from all outbreaks underwent necropsy and samples for microbial investigations were obtained from various organs (Table 2). Septicemia was the dominating finding in most chickens including lesions like fibrinous perihepatitis, pericarditis and peritonitis, and less frequently arthritis. Antimicrobial treatment was initiated in case 1–5, yet with limited curative effect. In case 6 and 7 no antimicrobial treatment was initiated. Instead, affected chickens were euthanized due to welfare reasons.

**Table 2:** **Characteristics of the seven outbreaks by** ***E. coli*** **in a broiler production unit.**

Outbreak	Date	Age (days)	Pathology	Chicken isolation site	No. of isolates	Antimicrobial treatment
1	Aug. 2017	5	Peritonitis	Liver	1	Florfenicol
2	Oct. 2017	14	Septicemia	Liver	5	Doxycycline
3	Dec. 2017	6	Septicemia	Liver	1	Doxycycline
4	Feb. 2018	18	Septicemia	Liver	5	Sulfa/trimethoprim
5	Jun. 2018	18	Septicemia	Liver	17	Doxycycline
6	Oct. 2018	18	Septicemia	Pericardium/hip joint/peritoneum	6	None
7	Nov. 2018	21	Septicemia	Liver (rat intestine)	2	None
Total					37	

A total of 37 bacterial isolates (36 from chickens and 1 from a rat).

To establish a microbial diagnosis, samples were obtained from the liver of dead chickens following necropsy at the farm using BD BBL™ CultureSwab™ Transport Systems (Fischer Scientific) and shipped to Department of Veterinary and Animal Sciences, University of Copenhagen. All swabs were obtained prior antibiotic use. The swabs were streaked onto blood agar plates (Blood agar base, CM55; Oxoid) added 5% bovine blood and incubated aerobically for 16 h. From all cases, pure cultures with at colony morphology resembling *E. coli* were found. A total of 37 bacterial isolates were obtained from the seven investigated outbreaks. The identity of each isolate was confirmed by qPCR [5].

**Figure 1:**
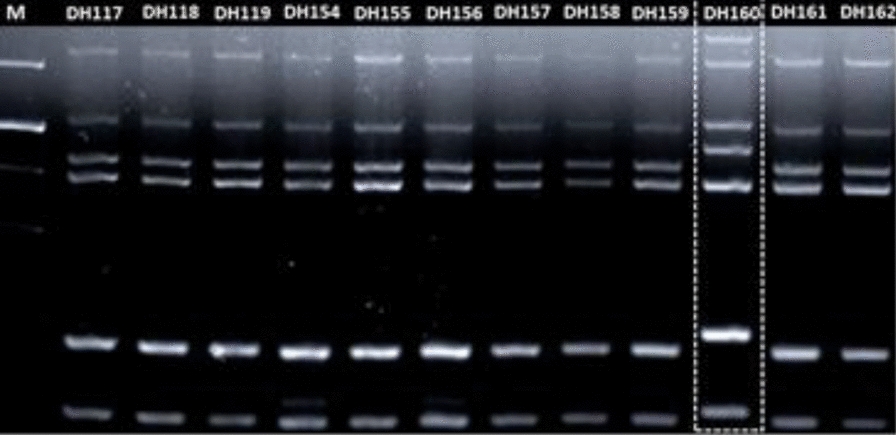
**Multiple-locus variable-number tandem-repeat analysis (MLVA) genotyping of 12 representative *E. coli* isolates originating from chickens of different rotations in the same broiler unit.** Isolate DH160 originated from the intestine of a rat euthanized in the unit.

Due to the recurrence of highly similar clinical signs and pathological manifestations, we investigated if the *E. coli* responsible for the outbreaks were linked or independent. For that purpose, we applied three different genotyping methods. First, all isolates were characterized by a multiple-locus variable-number tandem-repeat analysis (MLVA) as described by [6]. All but one isolate (DH160) had a highly similar banding pattern (Figure 1). The isolate with a deviating banding pattern originated from the intestine of at rat caught and euthanized in the broiler unit.

**Figure 2:**
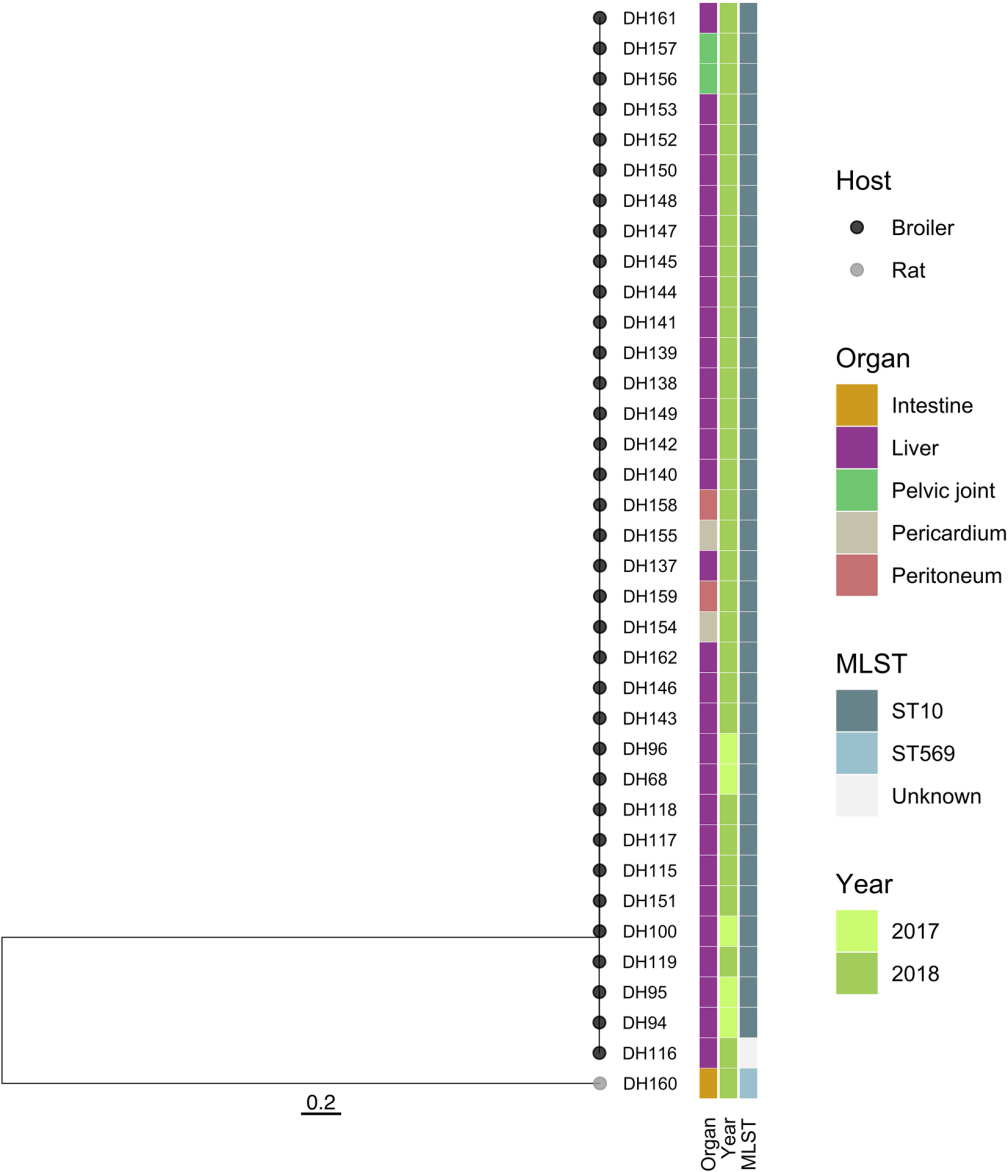
**Comparison of the core genomes of 37 *E. coli* isolates.** One isolate originated from a rat (DH160), while the remaining 36 isolates originated from different organs of broiler chickens reared during 2017 and 2018.

Secondly, all isolates were subjected to whole genome sequencing using the Illumina MiSeq System (Illumina, USA) and a 2 × 250 bp paired-end sequencing approach. Raw reads were quality-checked using FastQC 0.11.8 [7]. The FastQ files were uploaded to the EnteroBase *E. coli* database [3]. Core genome multi-locus sequence types and in silico serotype predictions were performed using the integrated tools in EnteroBase. All but the rat isolate (DH160) belonged to the ST10 sequence type, whereas the rat isolate belonged to ST569. Serotyping based on the H-type predictions for all ST10 isolates all predicted the H7 type, whereas for only a subset of the isolates the O-type could be predicted. In all cases the O-type was O132. Finally, to compare the isolates at an even higher resolution we performed core-genome single nucleotide polymorphism (SNP) analysis using the CSI Phylogeny 1.4 pipeline with default parameters [4]. Thirty-six (all ST10) of the 37 isolates were remarkably similar (Figure 2) and had a maximum of nine SNPs differences in their core-genome. The rat isolate differed with more than 15 000 SNPs from the remaining isolates (Additional file 1). The genotyping procedures indicated that the same *E. coli* clone caused all seven outbreaks during a course of 18 months. Prediction of virulence genes was performed using VirulenceFinder 2.0 [8] in five *E. coli* isolates, each representing an individual outbreak. All strains carried the same set of four virulence genes *cib* (colicin ib), *gad* (glutamate decarboxylase), *iss* (increased serum survival), and *terC* (Tellurium ion resistance protein). All detected genes were 98.98–100% identical to the corresponding template genes in the virulence database, including accession number KP198616 for *cib*, U00096 for *gad* (except strain DH95, with accession number CP002967), CP001509 for *iss* and CP007491 and MG591698 for *terC* (each strain displayed both *terC* matches).

In five of the seven outbreaks, antimicrobial treatments were initiated to reduce the negative health effects and mortality. Different antimicrobial compounds were used (Table 2) yet limited effect was observed. To obtain further insight into the antimicrobial susceptibility patterns, the same five *E. coli* isolates included in the analysis of virulence genes were characterized. The MIC was determined by broth microdilution according to the Clinical Laboratory Standards Institute [9] against 19 antimicrobial compounds. In addition, we submitted the genome sequence raw reads to ResFinder 4.0 in order to identify resistance genes [4]. The phenotypic and genotypic results are summarized in Table 3. Due to the repeated antimicrobial treatments, we wanted to investigate if any developments in the sensitivity profiles could be observed during the 18 months period. Briefly, antimicrobial resistance was observed towards ampicillin, doxycycline, tetracycline, trimethoprim/sulfamethoxazole for all five isolates tested. For one isolate (DH95) resistance was also observed towards chloramphenicol (MIC  > 32 mg/L) and the gene *floR* was identified in the genome sequence. Interestingly, the presence of this particular resistance phenotype appeared subsequent to florfenicol treatment of the rotation immediately preceding the one from which DH95 originated (Table 2).

**Table 3:** **Phenotypical and genotypical susceptibility profiles of five** ***E. coli*** **clones, each representing an outbreak.**

Antimicrobial	Strain	DH68	DH95	DH119 4	DH155	DH162	Resistance
	Case	1	2		6	7	Phenotype
Amikacin	MIC (mg/L)	≤ 4	≤ 4	8	8	≤ 4	Sensitive
	Genotype	–	–	–	–	–	
Amoxicillin/clavulanic acid	MIC	8	> 8	8	8	8	Sensitive
	Genotype	blaTEM-1B	blaTEM-1B	blaTEM-1B	blaTEM-1B	blaTEM-1B	
Ampicillin	MIC	> 8	> 8	> 8	> 8	> 8	Resistant
	Genotype	blaTEM-1B	blaTEM-1B	blaTEM-1B	blaTEM-1B	blaTEM-1B	
Cefalexin	MIC	4	8	4	4	4	Sensitive
	Genotype	–	–	–	–	–	
Cefazolin	MIC	4	4	4	4	4	Intermediate
	Genotype	–	–	–	–	–	
Cefovecin	MIC	0.5	1	0.25	0.25	0.5	Sensitive
	Genotype	–	–	–	–	–	
Cefpodoxime	MIC	≤ 1	≤ 1	≤ 1	≤ 1	≤ 1	Sensitive
	Genotype	–	–	–	–	–	
Ceftazidime	MIC	≤ 4	≤ 4	≤ 4	≤ 4	≤ 4	Sensitive
	Genotype	–	–	–	–	–	
Chloramphenicol	MIC	4	32	8	4	4	Sensitive/resistant
	Genotype	–	*floR*	–	–	–	
Doxycycline	MIC	> 8	> 8	> 8	> 8	> 8	Resistant
	Genotype	*tetA*	*tetA*	*tetA*	*tetA*	*tetA*	
Enrofloxacin	MIC	≤ 0.12	≤ 0.12	≤ 0.12	≤ 0.12	≤ 0.12	Sensitive
	Genotype	–	–	–	–	–	
Gentamicin	MIC	1	1	1	1	1	Sensitive
	Genotype	–	–	–	–	–	
Imipenem	MIC	≤ 1	≤ 1	≤ 1	≤ 1	≤ 1	Sensitive
	Genotype	–	–	–	–	–	
Marbofloxacin	MIC	≤ 0.12	≤ 0.12	≤ 0.12	≤ 0.12	≤ 0.12	Sensitive
	Genotype	–	–	–	–	–	
Orbifloxacin	MIC	≤ 1	≤ 1	≤ 1	≤ 1	≤ 1	Sensitive
	Genotype	–	–	–	–	–	
Piperacillin/tazobactam	MIC	≤ 8	≤ 8	≤ 8	≤ 8	≤ 8	Sensitive
	Genotype	blaTEM-1B	blaTEM-1B	blaTEM-1B	blaTEM-1B	blaTEM-1B	
Pradofloxacin	MIC	≤ 0.25	≤ 0.25	≤ 0.25	≤ 0.25	≤ 0.25	Sensitive
	Genotype	–	–	–	–	–	
Tetracycline	MIC	> 16	> 16	> 16	> 16	> 16	Resistant
	Genotype	*tetA*	*tetA*	*tetA*	*tetA*	*tetA*	
Trimethoprim/sulfamethoxazole	MIC	> 4	> 4	> 4	> 4	> 4	Resistant
	Genotype	*dfrA1/sul2*	*dfrA1/sul2*	*dfrA1/sul2*	*dfrA1/sul2*	*dfrA1/sul2*	

## Discussion

A maximum of nine SNPs differences in the core genomes of the characterized isolates indicates that the same *E. coli* clone was the main cause of the seven disease outbreaks in 11 consecutive rotations of the same broiler unit during an 18-month period. Accumulated mortality rates of up to 22% in the affected rotations underlined the severity of the situation. Interestingly, the problem both arose and disappeared suddenly. Vertical transmission was not regarded very likely as the affected chickens were generally older than typically seen for vertically transmitted *E. coli* and no difference in the 1st-week mortality was observed (Table 1). In addition, the responsible *E. coli* clone was not reported as being broadly involved in disease from other broiler farms receiving chickens from the same hatcheries during the period. On the contrary, *E. coli* ST10 was at the time an uncommon sequence type among Danish broilers. Focus was thus aimed at horizontal transfer, and meticulous revision of the cleaning and disinfection procedures was made at the farm. Cracks and crevices in the cement floor preventing complete cleaning and disinfection were identified and renovated. The main drinking water supply line was also in need of renovation and was renewed completely towards the end of the problematic period. Although the exact source of the responsible bacterium was not determined, environmental persistence despite rigorous cleaning and disinfections procedures points at a source within the broiler unit, which may have been the damaged floor, the feeding system and/or the water supply. *E. coli* has been demonstrated able of surviving prolonged periods outside the typical host environment [10].

The highly limited genetic difference in the *E. coli* outbreak clone core-genome indicated limited evolution of the clone despite infection of thousands of chickens. As no immunity towards the clone was build up due to the complete stop–go nature of the all-in-all-out system, the same clone appeared to get seeded into the flocks over and over again, possibly from the same source, where it very successfully infected the chickens and caused high mortality. Whether the recurring outbreaks replenished the source of infection or the source remained constant is unknown. *E. coli* ST10 belong to the global extraintestinal pathogenic *E. coli* lineages of increasing importance in human infections, yet the importance of this sequence type in poultry is less well investigated [11]. Clonal stability within *E. coli* sequence types has been described but only to a limited extend [12]. The genetic variation observed in the 18-month period is comparable to the 0–6 SNPs difference observed in within-host intraclonal *E. coli* isolates from human urinary tract infections and feces [13].

Similarly to the SNP analysis, the virulence profile was also consistent among the representatives of each outbreak. Interestingly, the virulence gene *cib* detected in our strains was 100% identical to that of the accession number KP198616, which is located on a CTX-M-123 carrying plasmid in *E. coli* ST10 isolated from poultry in Hong Kong [14]. Another virulence gene *iss*, has been associated to poultry colibacillosis before [15]. Neither the ESBL genotype or phenotype was, however, detected in the five strains characterized in the current investigation.

Another parameter of high importance for the clinical outcome is the antimicrobial susceptibility of the outbreak-associated isolates. Due to high relatedness of the outbreak isolates, we expected few differences in their resistance phenotypes. An obvious explanation for the limited success of the antimicrobial on-farm treatments during the outbreaks was the resistance phenotypes against doxycycline and trimethoprim/sulfamethoxazole, which were the compounds used in four out of the five treatments. In outbreak 1, the treatment was made with florfenicol, for which the outbreak clone was supposed to be susceptible. However, interestingly, the clone isolated during outbreak 2, the rotation immediately subsequent to the florfenicol treated rotation, had a MIC value of  > 32 mg/L and was thus resistant. Florfenicol resistance, here encoded by *floR*, has both been described as of chromosomal and plasmid origin [16]. The *E. coli* ST10 clones isolated from outbreak 3 and onwards were not resistant to florfenicol indicating that the genotype and associated phenotype apparently disappeared in the later outbreaks.

In conclusion, our investigation indicated that the same *E. coli* ST10 clone persisted in a broiler unit over an 18-month period. The *E. coli* clone was involved in seven outbreaks with highly elevated mortality despite antimicrobial treatments.

## Supplementary Information

**Additional file 1. Core genome comparisons of 37** ***E. coli*** **isolates and the number of single nucleotide polymorphisms (SNPs) between each isolate**. Isolate DH160 (isolated from a rat) differed from the remaining 36 chicken isolates with more than 15 000 SNPs, whereas a maximum of nine SNPs differences was observed between the chicken isolates.

## Data Availability

All sequence files have been submitted to the National Center for Biotechnology Information (NCBI) and are publicly available under BioProject Number PRJNA778644. All other material is available from the author of correspondence.
