# Supplementary figures and images for: Recurring outbreaks by the same Escherichia coli ST10 clone in a broiler unit during 18 months
Source: Vet Res. 2022 Jan 9;53:2. doi: 10.1186/s13567-021-01017-6 (PMC8744217; doi:10.1186/s13567-021-01017-6)

## Slide 1
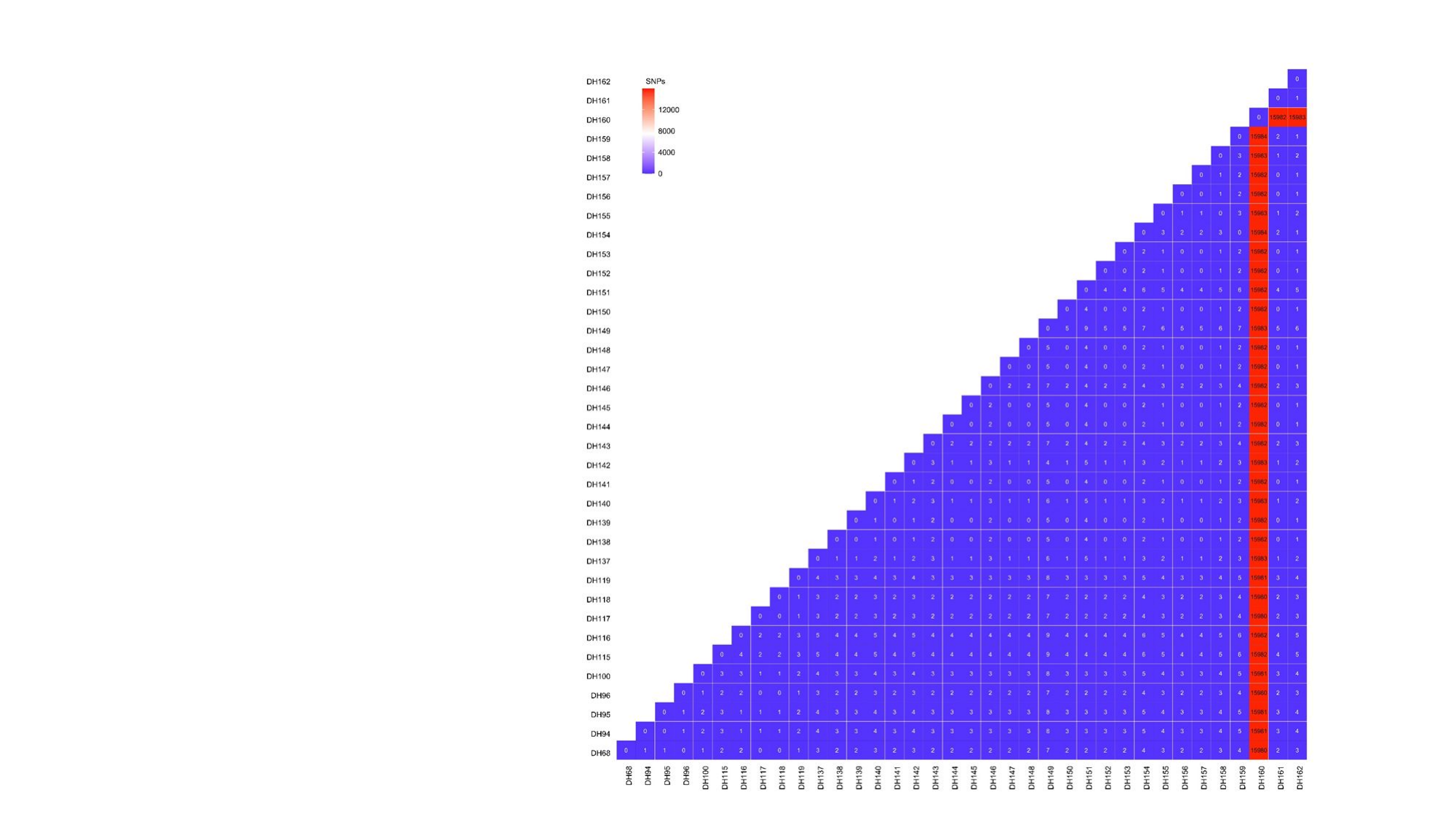

Supplement: Supplementary file 1 — Additional file 1. Core genome comparisons of 37 E. coli isolates and the number of single nucleotide polymorphisms (SNPs) between each isolate. Isolate DH160 (isolated from a rat) differed from the remaining 36 chicken isolates with more than 15 000 SNPs, whereas a maximum of nine SNPs differences was observed between the chicken isolates. [file 13567_2021_1017_MOESM1_ESM.pptx]
